# General Practice On-the-Job Training in Chinese Urban Community: A Qualitative Study on Needs and Challenges

**DOI:** 10.1371/journal.pone.0094301

**Published:** 2014-04-11

**Authors:** Yali Zhao, Rui Chen, Bo Wang, Tao Wu, Yafang Huang, Aimin Guo

**Affiliations:** 1 School of General Practice and Continuing Education, Capital Medical University, Beijing, China; 2 National Health and Family Planning Commission, Beijing, China; 3 Beijing An Zhen Hospital, Beijing, China; Supportive care, Early DIagnosis and Advanced disease (SEDA) research group, United Kingdom

## Abstract

**Background:**

On-the-job training is an important strategy for general practitioners to deliver appropriately community health services in China. The development of basic professional competence for general practitioners is the main goal of on-the-job training program. The aim of this study was to explore the needs of and the challenges to on-the-job training for general practitioners, and to provide advices for policy-makers to carry out this program more effectively.

**Methods:**

We conducted 3 nominal group techniques, 17 in-depth interviews and 3 focus groups to identify the status of, needs of and challenges to on-the-job training for general practitioners in Liaoning, Ningxia, and Fujian provinces from September 2011 until December 2011. Audiotapes and transcripts were analyzed to identify major themes. Content analysis of the data was completed from January 2012 to March 2012.

**Results:**

Basic theoretical knowledge and clinical skills were the main needs for general practitioners during on-the-job training. The challenges during training included the time contradiction between work and training, deficiencies of qualified preceptors, and lack of training funds. Participants gave recommendations how to resolve the above problems.

**Conclusions:**

In order to improve the outcomes of general practice on-the-job training, it is necessary for government officials to resolve the contradiction between work and training, train preceptors continuously, and increase financial support in the training program.

## Introduction

In order to lower the burden of health care expenses and promote to get access to health services, the Chinese government launched its community health service (CHS) program in 1997. At present, Chinese city CHS organizations include the two-level CHS centers and their affiliated stations [1], [2]. The CHS organizations consist of three categories of CHS models of ownership and management: (1) 36.5% of the CHS organizations is government-owned and-managed, (2) 35.7% of the CHS organizations is government-owned and hospital managed, and (3) 27.8% of the CHS organizations is privately owned and managed [3], [4]. The main roles of CHS organizations are providing continuing and comprehensive basic clinical services and public health services to community residents. The core providers in the CHS organizations are general practitioners (family physicians) (GPs), public health specialists, and community nurses [5]. GPs are medical practitioners with recognized generalist training experience and skills, who provide and co-ordinate comprehensive medical care for residents [6]. In China, GPs are required to deliver some basic public health services besides basic medical services. However, qualified GPs are in short supply [7].

Although the CHS system is established, the development of CHS is slow due to the lack of awareness in its importance and scarcity of a gate-keeping role of qualified CHS providers [8]. Patients naturally choose to see doctors in hospitals because of their higher prestige and perceived levels of training [2]. In the current competitive health service market, physicians with better educational backgrounds or higher professional titles prefer to work at hospitals. At the same time, the community physicians are mainly the ones who received a 3–5 year medical education, which cannot meet the high expectation of the patients. In order to adapt to the changes in the health service model and to be able to provide adequate CHS, a series of policies were promulgated to encourage medical schools and affiliated hospitals to train up qualified GPs through general practice education [9].

There are two models currently for GPs' training in China (Table 1). The first model is the postgraduate residency training program. In August 2011, the Chinese Government released a policy statement [10] which established the system of training GPs, and which emphasized a 3-year general practice postgraduate residency training program. The residency training program aims to develop professional competence of a physician, who can be ready to practice in the specialty of general practice. The second one is on-the-job training program that started from 2000. It involves training the majority of the less-educated physicians who currently work in local CHS organizations and transforming them into qualified GPs [5]. Before 2010, the on-the-job training program was the main measure in China [11].

**Table 1:** Differences and similarities between the postgraduate residency training program and the on-the-job training program*.

		Postgraduate residency training program	On-the-job training program
Differences	Target population	Graduates whose medical background is 5- to 8-year post–high school education or over, and who are likely to choose general practice as a career.	Community health physicians with 3- to 5-year post–high school medical education,who are willing to become general practitioners by on-the-job training.
	Teaching contents/curriculum	(1) Clinical rotation in teaching hospitals for 27 months, (2) practice-based training in community for 6 months, and (3) optional contents for 3 months	(1) An academic curriculum on basic sciences and theory of general practice in the first month, (2) the primary rotational blocks in teaching hospitals in the following 10 months, and (3) practice-based training in community in the last month.
	Years of lengths	36 months	12 months
	Type of certificates obtained	Standardized residency training certificate which is issued by National Health and Family Planning Commission and is a prerequisite for the qualifications of GPs.	GPs on-the-job training certificate which is issued by provincial health department.
Similarities		Making trainees become qualified GPs	Making trainees become qualified GPs

* Source: “General practitioners standardized training standards (trial implementation)” (2011) and “General practitioners on-the-job training syllabus” (2007) issued by National Health and Family Planning Commission.

**Figure 1:**
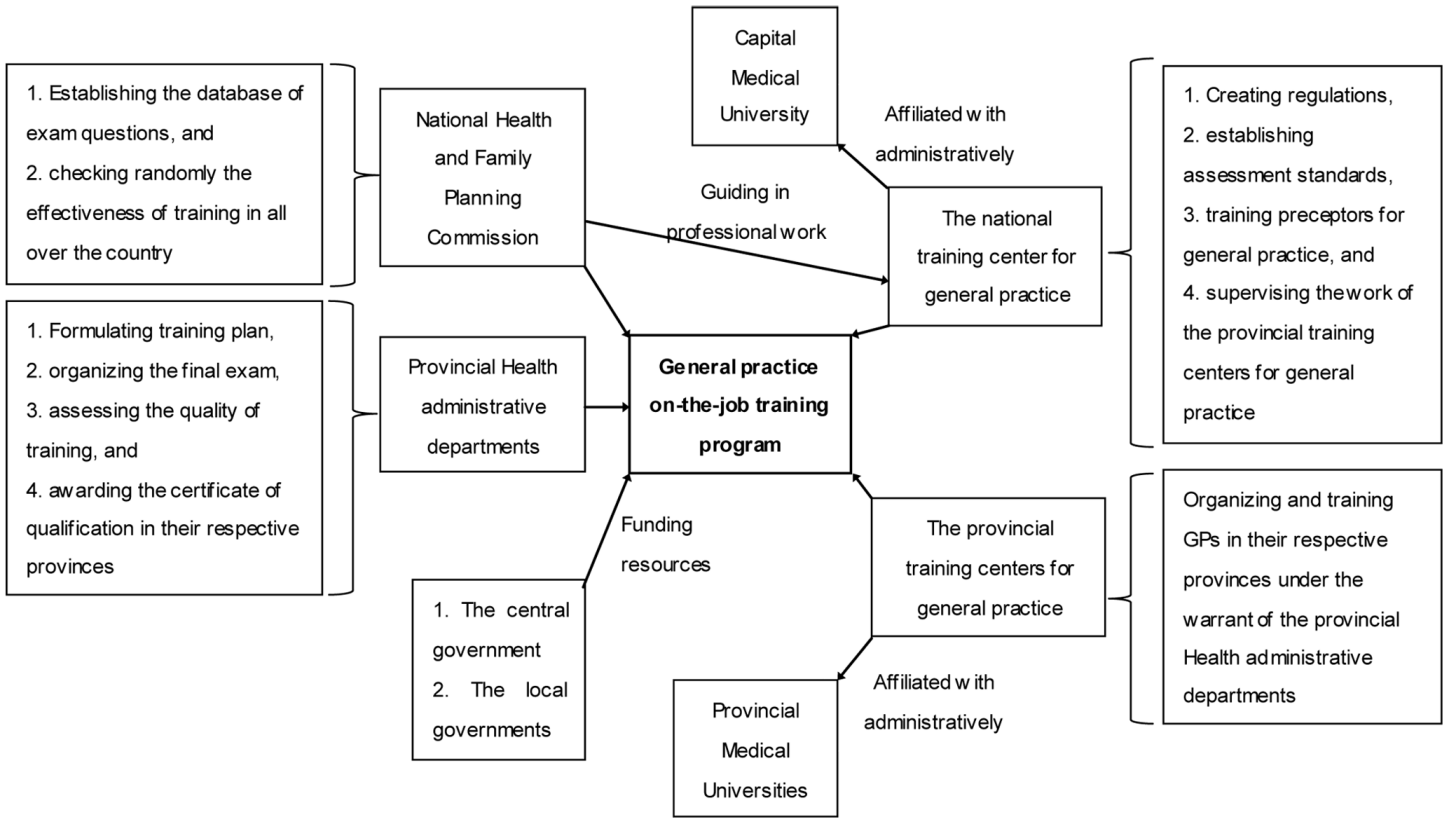
The framework for the different parties and organizations involved in general practice on-the-job training program. The different parties and organizations involved in general practice on-the-job training program include the national and provincial training centers for general practice, the health administrative departments from different provinces, and the central and local governments at all levels. Figure 1 describes the functions and roles of these parties and organizations on the training program, and helps readers to understand the background given the complicated healthcare and political system in China.

The national and provincial training centers for general practice are the organizations which manage and carry out the on-the-job training work. The national training center for general practice is affiliated with Capital Medical University administratively and is guided in professional work by the National Health and Family Planning Commission. The main responsibilities of the national training center for general practice include creating regulations, establishing assessment standards, training preceptors for general practice and supervising the work of the provincial training centers for general practice. All provincial training centers for general practice are in charge of organizing and training the community physicians in their respective provinces under the warrant of provincial health administrative departments. The health administrative departments from different provinces are in charge of formulating training plan, organizing the final exam, assessing the quality of training and awarding the certificate of qualification. The central and local governments at all levels are the providers of the costs for this training program (Figure 1).

At present, the average on-the-job training period is 12 months. Three phases constitute the whole training schedule: (1) An academic curriculum of basic sciences and theory of general practice in the first month, (2) the primary rotational blocks in teaching hospitals in the following 10 months, and (3) practice-based training in community in the last month.

The on-the-job training program for GPs has been implemented in China since 2000. To date, there has been no research on investigating the status quo and needs of on-the-job training for GPs in a national level. It is necessary to analyze and summarize the process of the project. A national questionnaire study had been carried out to attain the information about the status quo and the outcomes of the training. To determine ways to enhance future training work, we explored further the opinions and recommendations on training from trainees, administrators and preceptors by qualitative methods in this study built on the questionnaire study.

## Methods

### Sampling and Participants

The baseline questionnaire survey on the needs of and challenges to GPs training in the national urban CHS organizations was carried out during the period of September 2011–December 2011 by National Health and Family Planning Commission. Ten provinces including 30 administrative cities and 66 regions were selected by a multi-staged cluster sampling method in terms of economic characteristics, city size and the level of development of CHS.

The samples of this study included Liaoning, Ningxia, and Fujian provinces which were extracted from the above selected 10 provinces by three geographic regions according to economic statuses, developed, middle-developed and developing. We selected further three cities named Liaoyang, Yinchuan and Fuzhou from Liaoning, Ningxia and Fujian provinces respectively. The developments of CHS in three cities are mature and comparable. The CHS organizations in our study are of the same type across these three cities. They are government-own and -managed organizations. The participants were drawn from CHS organizations of the same types in the three cities. According to the inclusion criteria of participants and a sampling framework, a purposive sampling was adopted in each city (Table 2, 3). Two administrative staffs in the health bureau in each city, that were familiar with general practice training program and were trained before the study, were responsible for conducting sampling. Three types of people were recruited from each city above, named GPs, administrative officers and CHS organizations' directors on training, and preceptors in training GPs. The nominal group techniques (NGT) of GPs, the interviews of administrative officers and directors on training, and the focus groups of preceptors were implemented in each city, respectively. NGT represented the first stage and were used in an explorative manner to create a thematic framework of needs. During the second and the third stages, this proposed framework was tested in interviews and focus groups and further refined, and countermeasures were suggested to solve the challenges to on-the-job training in the last two stages.

**Table 2:** the inclusion criteria of GPs, preceptors and administrative staff from Liaoyang, Yinchuan, and Fuzhou cities.

Objects	Inclusion criteria
GPs	(1) Working experience in community for 5 years or over, (2) getting the certificate of general practice on-the-job training, and (3) willing to participate in the study.
Preceptors	(1) Teaching experience with general practice on-the-job training, (2) studying experience with training preceptor programs of general practice, and (3) willing to participate in the study.
Administrative staff	(1) Management experience with general practice on-the-job training, (2) studying experience with general practice management training, and (3) willing to participate in the study.

**Table 3:** Sampling framework of participants in Liaoyang, Yinchuan, and Fuzhou cities.

Objects	Item		Liaoyang	Yinchuan	Fuzhou
GPs	No. of government-owned and -managed CHS centers*		3	20	48
	No. of selected government-owned and -managed CHS centers		3	9	9
	No. of GPs from selected every CHS center		3	1	1
	No. of GPs in total		**9**	**9**	**9**
Preceptors	No. of training bases for general practice in total*		5	10	21
		No. of universities	0	1	2
		No. of teaching hospitals	4	7	9
		No. of CHS centers	1	2	10
	No. of selected general practice training bases		5	5	6
		No. of universities	0	1	2
		No. of teaching hospitals	4	3	3
		No. of CHS centers	1	1	1
	No. of preceptors in total		**6**	**6**	**6**
		No. of preceptors from selected universities	0	2	2
		No. of preceptors from selected teaching hospitals	4	3	3
		No. of preceptors from selected CHS centers	2	1	1
Administrative staff	No. of training bases for general practice in total*		5	10	21
		No. of universities	0	1	2
		No. of teaching hospitals	4	7	9
		No. of CHS centers	1	2	10
	No. of selected general practice training bases		2	4	5
		No. of teaching hospitals	1	2	2
		No. of CHS centers	1	2	3
	No. of interviewees in total		**4**	**6**	**7**
		No. of admin staffs from the provincial health department	1	1	1
		No. of admin staffs from the municipal health bureau	1	1	1
		No. of directors from selected teaching hospitals	1	2	2
		No. of directors from selected CHS centers	1	2	3

*: These figures were obtained from the internal data of the health bureaus of Liaoyang, Yinchuan, and Fuzhou cities in 2011.

In this study, we used the term ‘GPs’ synonymously with that of trainee and the term ‘preceptor’ to denote a general practice teacher or trainer.

### Data Collection

#### NGT

Three NGT were carried out in Liaoyang, Yinchuan, and Fuzhou cities respectively, and 9 GPs were recruited within each group by purposive sampling. The age of 27 GPs ranged 29–60 years (mean age = 41.4±8.0 years) (Table 4).

**Table 4:** Demographic characteristics of participants.

Objects	Category	Subcategory	N
GPs	Gender	female	20
		male	7
	Medical educational background	5–8-year post-high school program	9
		3-year post-high school program	16
		4-year post-middle school program	2
	Professional positions	senior-level title	2
		middle-level title	17
		junior-level title	8
Preceptors	Gender	female	11
		male	7
	Medical educational background	8-year post-high school program	6
		5-year post-high school program	12
	Professional positions	senior-level title	11
		middle-level title	7
	Occupations	university faculty member	4
		teaching hospital doctor	10
		CHS organization GPs	4
Administrative staff	Gender	female	12
		male	5
	Medical educational background	8-year post-high school program	2
		5-year post-high school program	10
		3-year post-high school program	5
	Professional positions	senior-level title	7
		middle-level title	8
		junior-level title	2
	Occupations	administrative officers from the health departments or bureaus	6
		directors from teaching hospitals	5
		directors from the CHS organizations	6

During NGT, our main point of interest was the identification and priority of training needs of GPs in theoretical knowledge and basic clinical skills. Two questions were formulated during NGT session: (1) what are the theoretical knowledge that you want to learn? (2) what are the basic clinical skills that you need to learn during training? The GPs started with a blank sheet and wrote three ideas for every question. Then the facilitator gathered the ideas of all group members and asked participants to prioritize the items. Each group members selected the three most important items from the group list, and then ranked the three ideas selected, with the most important receiving a rank of 3, and the least important receiving a rank of 1. NGT groups were conducted in the meeting rooms of the health bureaus in the three cities above. The facilitator is an assistant professor and has qualitative and social research experience in community-based medical researches. She chaired the meetings. A Ph. D. degree candidate with a general practice degree made records by using a computer during the meetings.

#### Semi-structured interviews

Semi-structured in-depth interviews were conducted with 17 participants who managed the GP training. The age of the interviewees ranged 30–50 years (mean age = 40.8±5.2 years) (Table 4). Trained professional interviewer implemented the interviews in the participant's offices from September 2011 to December 2011. The interviews lasted 60 to 90 minutes. All participants were informed about the purpose of the study and that they could stop the interview at any point without giving a reason. Written informed consent and an agreement that quotes from the interviews could be used anonymously were obtained from all participants. The interviewer, who is a professor and a director of school of general practice and continuing medical education, has engaged in research, education and training of general practice for twenty years. She directed the whole process of the interviews. A team member familiar with qualitative research took extensive notes, in addition to tape recording and transcribing the interviews. The interview questions were open-ended and covered issues about the status of and challenges to on-the-job training.

#### Focus groups

Three focus groups were organized, and one group was set in each city, and 6 preceptors were recruited in each group. Focus group participants were chosen for their knowledge of, preceptor training experience with, and interest in on-the-job training. The age of the preceptors ranged 29–51 years (mean age = 42±6.8 years) (Table 4). The participants discussed the needs of and challenges to on-the-job training for GPs to identify potential ways to address the challenges.

Focus groups sessions, lasting 90 minutes, were facilitated by the lead researcher, and were audio-taped with permission. 3 focus groups were conducted in the meeting rooms of the health bureaus in the three cities above. Participation was voluntary, and subjects were free to withdraw at any stage without giving a reason. Ethical issues concerning the confidentiality of participants were highlighted. The facilitator is an expert in community medicine. At the same time, he is very familiar with health policy and training for general practice. One study member documented the discussion by taking notes. The summaries of 3 focus groups were created by reviewing the audiotapes and combining the notes.

### Ethics Statement

This study was approved by the Medical Ethics Committee of Capital Medical University, Beijing, China. Anonymity and informed consent were guaranteed prior to participating in the study, and no sources of potential harm to the participants were apparent. In line with the terms of consent to which participants agreed, the personal details data were not publicly available other than age, gender, medical educational background, professional position; these data were gathered on consent forms.

### Data Analysis

Our research team reviewed the transcripts. We reached consensus in analysis and interpretation by using an iterative process in meetings. Our research team was a multidisciplinary group including 2 community-based medical researchers with qualitative and social research experience, 1 health administrator from a health department who is familiar with health policy, 1 researcher familiar with medical education, 1 epidemiologist and 1 Ph. D. degree candidate with a general practice degree. The variety of perspectives of our team analyzed the data in order to maintain the validity and meaningfulness of the results.

Qualitative content analysis [12] approach was used to analyze the interviews and focus groups' data from January 2012 to March 2012. The team members read the transcripts through several times and then independently coded transcripts. These codes were identified between two researchers and used to revise the thematic framework. A consensus of the members resolved coding differences after thorough discussion in order to make sure all perspectives on the themes were represented in the written results. The themes that emerged for the purposes of this report included the content of the needs and challenges to on-the-job training.

The data was collected and analyzed during NGT according to the procedure of NGT: (1) the facilitator presented the question; (2) the GPs generated three responses on a blank sheet independently and silently; (3) the facilitator used a round-robin approach to allow each GP to read out an item from their list; (4) each GP clarified his/her items clearly, and similar items were combined under one heading; (5) the facilitator collected all items, entered them into a database established in Excel 2007, and displayed them on the screening; (6) each GP was asked individually and confidentially to rank the three items, with the most important receiving a rank of 3, and the least important receiving a rank of 1, from the full list of items on the screening; (7) the facilitator collected all votes and assigned votes to each item and their rank order which were shown on the screening; (8) the facilitator asked GPs to raise further issues about the items to reach consensus agreement.

## Results

The 4 themes emerged and included GPs' training needs, effects of the training program, challenges in providing training, and solutions to overcome the challenges. Basic knowledge and basic skills presented needs of GPs. The participants analyzed the effect and barriers occurred during on-the-job training. Additionally, interviews and focus groups identified 3 advices to overcome challenges to enhancing quality of training: (1) flexibility of training time, (2) continuity of preceptors learning, and (3) supplying sufficient funding for training.

### GPs' training needs

During NGT, the GPs pointed out the important theoretical knowledge and basic clinical skills which they wanted to learn during training (Table 5–10). The important theoretical knowledge included mainly the diagnosis and treatment of the common diseases, and the systematic management of chronic diseases. The basic skills mostly were involved: (1) the film reading of CT, X, MRI, ECG and Ultrasound, (2) physical examination, and (3) the techniques of emergency and first aid.

**Table 5:** Theoretical knowledge which general practitioners want to learn during training by NGTs in Liaoyang city.

No.	Item	Scores
1	Theoretical knowledge of common diseases in community	**11**
2	Preferred medication of common diseases in community	**10**
3	The health education of chronic diseases in community	**8**
4	The knowledge of dermatology	5
5	Diagnosis of CT and X-ray	5
6	The knowledge of emergency and first aid	4
7	Chronic diseases management	3
8	Theoretical knowledge of laboratory tests	2
9	Theoretical knowledge of surgical operations	1
10	Theoretical knowledge of infusion reactions	1
11	Theoretical knowledge of TCM*	1
12	Common diseases of gynecology	1
13	Cardiovascular diseases	1
14	Clinical basic theory	1
15	Child care	0

*: TCM = Traditional Chinese Medicine.

**Table 6:** Basic clinical skills which general practitioners want to learn during training by NGTs in Liaoyang city.

No.	Item	Scores
1	The film reading of CT, X, MRI and Ultrasound	**9**
2	Emergency treatment of infusion reaction	**8**
3	Physical examination	**7**
4	Surgical treatment of common health problems	6
5	The reading of laboratory tests	6
6	The reading of ECG	5
7	Emergency and first aid	5
8	Treatment on adverse reactions of vaccines	3
9	Treatment of allergic reactions	2
10	Surgical skills	2
11	The use of ultrasound therapy instruments	1
12	Acupuncture and Cupping	0
13	Palpation and percussion skills	0

**Table 7:** Theoretical knowledge which general practitioners want to learn during training by NGTs in Yingchuan city.

No.	Item	Scores
1	Theoretical knowledge of TCM* and common diseases in community	**7**
2	Systematic management of diabetes and hypertension	**7**
3	Immunization and maternal/child care	**6**
4	Diagnosis and treatment of pediatric diseases	4
5	The knowledge of emergency and first aid	4
6	The philosophy of general practice	3
7	The differential diagnosis of common diseases	3
8	The prevention and treatment of chronic diseases	3
9	Community-based rehabilitation	3
10	The knowledge of preventive healthcare	2
11	The standard medication of respiratory diseases	2
12	The diagnose and treatment of the common diseases in gynecology	2
13	Elderly care	1
14	Psychological counseling and treatment	1
15	The knowledge of dermatology	0

*: TCM = Traditional Chinese Medicine.

**Table 8:** Basic clinical skills which general practitioners want to learn during training by NGTs in Yingchuan city.

No.	Item	Scores
1	Appropriate technology of TCM	**14**
2	Operation and reading of ECG	**9**
3	Emergency treatment	**8**
4	The handle of wound	5
5	The skills of health education	5
6	Radiation	4
7	B ultrasound	3
8	The differential diagnosis of common diseases in children	2
9	Physical examination	2
10	Routine blood tests	1
11	Routine urine testing	1
12	Stethoscope	0

**Table 9:** Theoretical knowledge which general practitioners want to learn during training by NGTs in Fuzhou city.

No.	Item	Scores
1	Principles of diagnosis and treatment of chronic diseases	**9**
2	Emergency and first aid	**8**
3	Theoretical knowledge of common diseases in clinic	**6**
4	The knowledge of community management	4
5	Infectious diseases screening	3
6	Application of laboratory tests	2
7	Psychology	2
8	Outpatient management	2
9	Synergy and antagonism between drugs	2
10	Rational use of antibiotic	1
11	The health education and following-up of common diseases	1

**Table 10:** Basic clinical skills which general practitioners want to learn during training by NGTs in Fuzhou city.

No.	Item	Scores
1	Physical exam	**12**
2	The film reading of CT, X, MRI, ECG and Ultrasound	**9**
3	Techniques of emergency and first aid	**7**
4	The basic clinical skills in community	5
5	Doctor-patient communication skills	3
6	Community-based rehabilitation techniques	3
7	The use of non-invasive ventilation	2
8	The establish and management of health record	1
9	Punctures skills	1
10	The methods of health education	1

The interviews and focus groups identified the basic knowledge and basic skills as centrally important during training. Participants believed strongly that the successful training programs should attach importance to the needs of trainees, and the preceptors from teaching hospitals should pay attention to teaching of basic skills.

“*General practice is the point of first contact for the majority of people seeking health care…, GPs must grasp basic knowledge and skills to deal with common problems (CHS organization preceptor 2).” “During rotation blocks, teaching hospitals should train GPs to grasp the basic skills such as emergency treatment, physical examination, and aseptic operation (Administrative officer 2).*”

### Effects of the training program

The participants agreed that the quality of GPs' education and patients' medical care were greatly improved by on-the-job training. They identified that CHS organizations benefited from the community-based training in the improvement of training equipments and the enhancing of service capabilities. There was a marked increase in the cooperation and communication between teaching hospitals and CHS organizations because of the training program.

*“The higher level of patient care and greater satisfactions of residents to CHS were reported because the service capabilities of GPs were improved by training (Teaching hospital director 1).” “After training, a good interactive relationship was built between our hospital and the communities where some trainees came from, and we supplied some technical support to them in treating patients (Teaching hospital preceptor 3).”*

### Challenges in providing training

The participants conveyed the opinion that some challenges remained to the training.

#### Time contradiction between work and training

Besides supplying medical care, GPs are required to delivery cost free clinical preventive services for individuals and families and population-based public health services. In term of service population, the quantity of medical staff in community sites is inadequate seriously. There was consensus among the participants that time contradiction between work and training was the most main challenge to GPs. It is very difficult for CHS organizations to dispatch medical staff out for training.

*“Trainees felt that they were under great stress and harried by many competing demands for their time (CHS organization director 2).”*

#### The deficiency of qualified preceptors

Qualified preceptor is one of the key factors to train qualified GPs. For becoming general practice preceptors, specialists from teaching hospitals must be trained to learn the knowledge of general practice for 10 days. The main training contents include the principles and competencies of general practice, the role of specialists in general practice education, and teaching critical thinking. Meanwhile, some senior GPs will become preceptors in community after training. But most of participants in interviews and focus groups remarked that the level of general practice teaching still mismatched trainees' need.

*“A specialist or a* GP *is impossible to become a qualified preceptor of general practice only through a short-term training (Administer officer 5).” “During clinical internship, most of preceptors in hospitals still follow the biomedical model with teaching (Teaching hospital preceptor 4).”*

#### Lack of funds

The central government provides a certain amount of funds to each province for on-the-job training of general practice. Accordingly, each local government was required to supply different amounts of money in its province up to its economic level and the number of trainees. The participants commended that the funding for training was insufficient from local support and that most of the funds were paid to the consumption of correlative public equipment and trainees' accommodation. Few financial incentives are paid to the individual preceptor or trainees as an encourage means.

*“Funding for on-the-job training from local governments should be invested timely (Administrative officer 1).” “No exact budget support for individual may be an important reason why we can't motivate trainees to take part in training initiatively (CHS organization director 1).” “When GPs in community stop working and participate in training for 12 months, their salary will be cut in half. To avoid this from happening, most of GPs refuse to participate in the training, or have to go back to work during the training (Administrative officer 3).”*

### Solutions to overcome the challenges

#### Flexibility of training time

Flexibility of training time was recommended as an effective method to resolve the contradiction between work and training. Most of the participants acknowledged that it would be feasible to divide the whole theory schedule into different module courses. Another feasible solution advised by all participants was to carry out the asynchronous online courses for trainees from different CHS organizations.

*“Trainees will have more flexible opportunities and time to accomplish the whole training courses by internet (CHS preceptor 1).”*

#### Continuity of preceptors learning

In order to becoming qualified, preceptors need to learn and practice the professional knowledge of general practice and teaching skills continuously. More training bases should be established to promote training in district and county areas, and the supervising of training preceptor programs is essential. Most of participants believed that regular training and assessment to preceptors were important to ensure the quality of preceptors.

*“Systematic network training is a feasible method which promotes the continuity of preceptors training (University preceptor 2).” “In order to improve the level of teaching in general practice, preceptors from teaching hospitals should work in community for a short time every year, and be familiar with the operating mode of CHS (CHS organization preceptor 2).”*

#### Supplying sufficient funding for training

Regarding financial support, the participants replied that the local governments should supply more funding support for training. For encouraging preceptors or GPs to participate in continuing medical education programs more actively, more financial incentives need to be paid to individuals.

*“Encouraging and rewarding is a feasible method to attract preceptors or trainees to take part in training more actively (Teaching hospital preceptor 1).”*

## Discussion

Compared to the continuing improvement of primary healthcare facilities, the low levels of CHS providers' knowledge and skills is still a major problem in China [13]. The capacity of GPs to provide medical services was inadequate and did not match patients' expectations [14]. General practice on-the-job training program that started in 2000 is one of the solutions resolving the above problem. Now, it is time to assess how general practitioners have been taught traditionally. Much valuable information of on-the-job training was found via the qualitative study.

Some common reasons resulted in the similarity of the GPs' needs from three cities in our study: (1) because of the deficiency of traditional gate-keeping role of CHS organizations, patients prefer to see clinicians at hospitals rather than at CHS organizations for an authoritative diagnosis or treatment, which makes GPs be unable to enrich their clinical experience by contacting frequently with patients; (2) the majority of the GPs are less-educated, and their professional skills are inadequate; (3) during on-the-job training in hospitals, trainees were arranged to rotate in many different blocks and to confront a variety of diseases within 10 months, which made them have no much time to study deeply in the knowledge of common diseases and basic clinical skills.

The physicians at hospitals often diagnose diseases by means of advanced instruments and equipment, while some advanced equipment are not provided in CHS organizations. When patients with a stable phase of diseases return to community from hospitals, they often consult GPs about the results of CT, X, MRI, ECG and Ultrasound from hospitals. Similarly, GPs are required to be capable of delivering the service of pre-hospital emergency care in community. In order to meet these expectations of the patients, GPs need to enhance corresponding knowledge and techniques by training.

There are five parts in the syllabus of general practice on-the-job training which include the basic knowledge of general practice, primary medical care, community prevention, community care and rehabilitation, and optional content (Table 11). Trainees were required to learn a lot of knowledge in a short time according to the syllabus of on-the-job training, which resulted in training contents not keeping pace with key desires of GPs. The result of our study demonstrated this conclusion. Besides the same needs of training with the other two cities, GPs from Yinchuan city presented particular demands of learning the knowledge and technology of Traditional Chinese Medicine. It suggested that training contents should be determined according to the actual needs of GPs in different regions in the process of improving training syllabus. Some studies [15], [16] indicated that adding more theoretical knowledge and basic clinical skills to the syllabus is an imperative task in improving training quality. Our findings are consistent with the researches [17], [18] that point out that the training program for GPs should focus on more tailored courses of doctor-patient relationship, psychosocial issues in medicine, patient physical diagnosis and interviewing, and basic clinical skills. The correct information provided to trainees is an appropriate response to their “need to know” [19].

**Table 11:** The content of general practitioners on-the-job training syllabus*.

Content	Item	Class hours
The basic knowledge of general practice	The conception and principles of general practice	56
	Community health service management and model	
	The establishing of health records	
	Management of health information system in community	
	Doctor-patient community skill	
	Family tree and family life cycle	
	Dual referral	
	Health management	
	The responsibility of general practitioners	
	Health policy and regulations of community health service	
Primary medical care	The methods of history collection and the establishing of medical record	328
	Physical examination	
	Diagnosis and treatment of common diseases and common symptoms	
	Prevention and treatment of infective diseases	
	Basic knowledge and treatment of emergency	
	Use of medicine	
	Using principles and reading of assisted examination methods	
	General surgical techniques	
	Diagnosis and treatment of pediatric common diseases	
	Diagnosis and treatment of gynecological common diseases	
	Prevention of Iatrogenic diseases	
Community prevention	Primary prevention, secondary prevention and tertiary prevention	60
	Community health assessment	
	Risk factors evaluation	
	Clinical prevention	
	Health education	
	Response to emergent public health hazards	
	Statistics and epidemiology in community	
Community care and rehabilitation	Child care	56
	Women's care	
	Elderly care	
	Rehabilitation in community	
Optional content	Laws and regulations in community health service	100
	Community health service management	
	Health economics in community	

*Source: Office of Ministry of Health (2007) “The notice of general practitioners on-the-job training syllabus (No. 48 document)”.

Because of heavy workload and unreasonable training time, GPs found it was difficult to make a rational arrangement between work and training. The low compliance of learning led to poor training effect. Some studies [20], [21] demonstrated that although the professional skills capability of trainees could be improved by training, the attitudes and behaviors of GPs in training were passive due to the lack of effective incentive and restraint mechanisms of learning. Our study suggested that it was difficult for GPs to make suitable arrangements between work and training, and the distant education via networks was a feasible training method during training. By creating a web-based resource for trainees to use, a consistent framework for trainees can be provided to guide their learning and develop competence in the broad scope of the practice [22]. A study [23] in Fujian province verified and supported our view that the contradiction between work and training can be settled down by on-line training for general practitioners.

The document in 2011 [24] mandated that the contents of on-the-job training will be determined according to the actual needs of GPs, and the whole training process will be divided into different phases based on the actual work time of GPs in different areas.

A high-quality general practice preceptor plays an important role in promoting effective implementation of general practice on-the-job training program. The preceptor is a facilitator who helps the trainee to learn new knowledge, attitudes, and skills. At present, qualified general practice preceptors are inadequate seriously [25]. It is difficult to train a qualified preceptor under the condition of deficiencies of training time and training practicality [26]. Establishing training centers for general practice preceptor in provinces and strengthening the construction of teaching staff are the key approaches to promote the development of general practice on-the-job training [27]. Meanwhile, sustainable fund investment from governments is an important measure to resolve the above dilemmas, especially in promoting the rewards of teaching [28].

### Strengths and Limitations

The strengths of this study are that it has brought in-depth investigation on the needs of and challenges to on-the-job training of general practice in a national level. We carried out the NGT, interviews and focus groups in this study, and a multidisciplinary team ensured a depth of understanding critical to the design of the study and the validity of results. The external validity was enhanced by purposeful sampling. However, 3 limitations need to be considered: (1) the data was cross-sectional in nature. It is possible that the other two categories of CHS models of ownership and management were different from government-owned and-managed CHS organizations in these three cities. The study did not examine the broader frame of overall organizations levels; (2) the data was collected in three provinces which may call into the question its generalizability to other locales; (3) during focus group, the preceptors in each group were not homogenous. Accordingly, further qualitative researches are needed on general practice on-the-job training with larger sampling from other cities in China.

## Conclusions

The results of our study demonstrated the effectiveness of on-the-job training program of general practice, reveal trainees' expectations, and elicit training's barriers. It is important that content of basic clinical knowledge and skills should be strengthened in training, and the flexibility of training time for trainees need to be resolved, preceptors' training should be continuous, and sufficient training funding must be provided. We believe it will require major reforms of on-the-job training of general practice in China to address these problems.
